# Why Multi-Family Groups for Transgender Adolescents and Their Parents?

**DOI:** 10.3389/fsoc.2020.628047

**Published:** 2021-01-25

**Authors:** Nicolas S. Rabain

**Affiliations:** Centre de Recherches Psychanalyse, Médecine et Société, University of Paris, Paris, France

**Keywords:** multi-family groups, contemporary families, innovating mental health care, trans identity, family relation, social adapation, same-sex parenting, psychological outcomes

## Abstract

The author presents a therapeutic approach for contemporary families carried out in an innovative mental health care setting. This approach involves receiving both transgender adolescents and their parents. Initially, the adolescents are brought together in a discussion group. Then, the parents of these adolescents are gathered without their children in order to reflect on family relations and social adaptation and to receive support when needed. Both groups are gathered together a couple of times a year in a multi-family meeting. Using a dynamic based on the principle of free association and the interplay of identifications among participants, the author points out how these groups and multi-family gatherings promote mental health and prevent mental disorders and the disruption of family relations.

## Introduction

In the context of contemporary family configurations such as same-sex parenting or single parenting, clinicians have been developing new and innovative approaches for providing therapeutic support in mental health care. Some are based on working with patients individually, while others favor a group approach, using focus groups, discussion groups or multi-family groups in which people accompanied by their children or their parents. The innovative mental health care setting we will present in this paper is meant for both transgender adolescents and their parents. Initially, the adolescents are brought together in a discussion group whose dynamic is based on the principle of free association. Then, their parents are received without their child in order to foster reflection regarding family relations and social adaptation as well as to receive support when needed. Both groups meet for a multi-family session two or three times a year in order to both promote therapeutic outcomes in case of psychological suffering or difficulties in family relationships, and prevent the disruption of family relations. In other words, this three-step approach aims at fostering a discussion about the challenges encountered by transgender adolescents and their parents regarding their family relations and social adaptation, and more particularly the difficulties faced in experiences of discrimination and rejection.

Who are our groups for and how do they function? How do they promote mental health? How do they prevent mental disorders?

## Materials and Methods

Ethical review and approval were not required for this study on human participants in accordance with the local legislation and international requirements. The participants provided their written informed consent before participating in this study.

In 2013, Professor David Cohen set up a Sexual Identity Consultation Service in his Child and Adolescent Psychiatry Department (La Salpêtrière Hospital, Paris). These types of consultations first emerged in the United States and Canada in the 1970s and eventually reached Europe through the Netherlands and the United Kingdom. In 2013, three pioneering consultation services were set up in France, including our own. Their common goal was to welcome people who presented “gender dysphoria”. Despite having a large cohort compared to other French hospitals, a statistic analysis remains impossible for the moment: in six years, our Sexual Identity Consultation only received about 150 transgender children and adolescents – which is definitely not enough for a statistic analysis. These figures demonstrate the lack of resources deployed across the country to support transgender minors (Condat et al., 2016; Mendès et al., 2016).

In our Child and Adolescent Psychiatry Department, most transgender teenagers seek our help in order to be guided through their transition. The initial request is often made with regard to the medical aspects of this process. Each month within La Salpêtrière Hospital, Multidisciplinary Concertation Meetings (MCM) bring together various professions: child psychiatrists, psychologists, pediatricians, psychomotor therapists, endocrinologists, reproductive biologists, ethicists and jurists. In this setting, they share their respective points of view and work out the handling of the most complex cases together. For instance, they can evaluate and question the relevance of prescribing a hormone therapy or a puberty blocker. They can also determine which treatment is likely to work best for which patient. In these meetings, decisions are made on a case-by-case basis.

In 2017, Professor David Cohen proposed me to create a new mental health care setting with Dr. Julie Brunelle in his Sexual Identity Consultation Service. Dr. Julie Brunelle had been working with transgender minors for several years whereas I was invited to participate as a therapeutic group specialist. As a matter of fact, I had introduced two years before a pioneer multi-family analytical group in *La Salpêtrière* Hospital for the attention of addicted teenagers. The setting-up of that group was based on my clinical background in the *Dr. Ricardo Gutiérrez* Hospital for Children in Buenos Aires. Argentina is the country where multi-family psychoanalysis was created in the 1960s by García Badaracco (1989) and García Badaracco (2000). This new setting was initially for the attention of schizophrenic patients but its indications have become more and more diverse. Nowadays, it can be indicated for young patients who have so far been resistant to traditional therapies. They usually suffer from behavioral disorders, eating disorders, addictions, narcissistic issues, an anxio-depressive syndrome or separation anxiety (Rabain and Bourvis, 2016). Multi-family groups bring together *“between two and twenty families to exchange their stories, experiences and emotions. The sessions can last between 1 h and two and a half hours, depending on the number of participants, the types of pathology and the institutional framework. Communication is coordinated by a group of co-therapists who have been trained in individual, group and family psychotherapy techniques* […]*”.*

That’s how Dr. Julie Brunelle and I started bringing together in a group about 10 adolescents aged 13–19 who had questions regarding trans identity. At that time, we were one of the only Child and Adolescent Psychiatry Departments to offer group outpatient care to transgender minors in France. That group still operates today. Each session consists of two parts: first the reception of the ten or so transgender teenagers and then, separately, the reception of their parents. Both subgroups are “open,” which means that newcomers can be included along the way, and both are gathered together a couple of times a year in a multi-family meeting. We would like to point out that all participants are extremely regular, regardless of whether or not they come from Paris and its suburbs. In fact, some of them live in the country-side, in other cities, or in small villages and travel each month across the country to reach Paris, just because they do not want to miss a single group session! 50% percent of our patients come from outside of Paris area. Furthermore, we receive both families with privileged backgrounds as well as socially disadvantaged families.

From the very first meeting, we make it clear that our objective is to accompany the adolescents in the process of constructing their gender identity and to encourage the unfolding of their subjectivity in a context removed from gender stereotypes. Our objective with the parents is to reflect on family relations and dealing with all kinds of experiences of discrimination. Our group’s framework is founded on several rules: as soon as a new transgender teenager is included, everyone introduces themselves with their new name, their age, sometimes their class, musical tastes and extracurricular activities, just as they would in any other group of teenagers. They also ask if that new person wants to be referred to as “he” or “she”. Then, three fundamental rules are formulated at the outset. Firstly, *“Everything that is said in the group must remain there.”* This rule of *confidentiality* is extremely important. Secondly, *“Do not judge any member of the group!”* We call this the rule of *respect*. And finally, *“Ask for the floor when you feel the need or desire to speak… And speak up as much as you can!”.* Indeed, each person is invited to formulate what they want, what they feel, and what they need to say or question so that their words resonate with the others. This is the rule of *free association*. In other words, we do not work with a selection of topics prepared in advance.

As with the adolescents, the parent group works with the same rules (*confidentiality, respect* and *free association*). When a new mother or father is included, everyone also introduces themselves with their own name and their child’s. Moreover, the co-therapists comments aim to relaunch the associativity of the participants and do not consist in a direct interpretation such as in a psychoanalytic group. However, like any therapeutic framework, the group of transgender adolescents and that of their parents respond to the five criteria of Racamier (2002): 1) a fixed *place* of the institution; 2) a fixed *period of time* (in this case, 1 h with the teenagers followed by 1 h with their parents) and a *frequency* (monthly meetings); the multi-family sessions are scheduled twice or thrice a year, last 1 h and a half with everyone together; 3) a substantial *number of families* under the guidance of two co-therapists and a secretary; 4) the three fundamental *rules* we mentioned above concerning all participants; 5) and, last but not least, paying attention to *border effects*, in other words the boundaries between the inside and the outside of the group, or even those intermediate moments when the session starts or ends.

After each session, both co-therapists and secretary have a half-an-hour discussion in order to talk about the group dynamics and the therapeutic process of each patient. That work of elaboration is based not only on the topics discussed by the patients and their emotions but also on our countertransference reactions. Moreover, every 2 months, we have a supervision with a private clinical psychologist in order to go on elaborating our movements of countertransference, from the notes taken by the secretary during the sessions and from what we remember. Once again, the dynamic is based on the principle of free association. In short, we are two co-therapists and one secretary presenting the clinical material from different perspectives to a psychologist who doesn’t work in our hospital but who has a significant experience of therapeutic groups.

## Results

During the first sessions, the most recurrent question usually concerns the hostility of the adolescent's environment, in particular the school environment and the neighborhood, or even hostility from relatives – frequently relatives outside the nuclear family. Most of the time, this phenomenon of rejection constitutes an impediment to the transition process. Nevertheless, this theme is unifying for the transgender teenagers as it allows them to temporarily set aside their differences: *“The other day, transphobes showed up in front of my school. They brought baseball bats!”*, says Jenifer. The place most often mentioned for being threatened or molested is school. David is indignant*: “In my college, the cisgenders sometimes dare to ask us what’s between our legs... We would never allow ourselves to ask them such intimate questions!”.* When speaking about cisgenders, intimacy is a theme that returns regularly as does the gaze of cisgender people: *“Some ‘cis-’people want to essentialize us; sometimes they try to sleep with us, just for one night, just to see, as though we were circus freaks!”.* Another unifying theme is sports, in particular the changing rooms, the swimming pool and summers at the beach: *“What if my binder shows under my T-shirt? What if I run into someone who knows my old identity…?”.* In short, recurrent themes in the adolescent group are hostility of the adolescent’s environment, intimacy and their involvement in an activist movement. Thanks to those common topics, the envelopes of the group are gradually being consolidated and the teenagers start to speak up: *“I was really scared before I came out; and I was a bit relieved afterwards; but as a matter of fact, I can only say this kind of thing here, in this group,* because I trust you guys!*”.*

In our experience, after about one year, opinions start to differ. At the same time, there is a reinforcement of the associative capacities of the participants and their capacity for contradiction. The following is an example of participants speaking up when they disagree: *“Should one say or hide one’s trans identity?”* For some, not everyone has to know: *“I don’t want to be a trans boy: I just want to be a boy!”*. Moreover, there is no need to inform people they meet after the transition. For others, the announcement makes it possible to sort out the people one trusts and those one will have to get rid of. There are also other differences: should one be an activist or not? Some reject the radical and sometimes caricatural style of LGBT activists: *“Only sectarian people expose themselves during the Gay Pride march… It hurts the cause! I won’t participate this year!”.* Others consider Gay Pride as *“…the only day of the year when we are not afraid, because we feel surrounded by our ‘new family’!”.*

Very often, transgender adolescents temporarily become the teachers of their own parents. The latter have not only subjective knowledge that inhabits their bodies, but also information gathered from social networks and blogs by transgender teens. The collective discourse is based on a lexical field of neologisms that they have to transmit to their parents. Transgender adolescents are also better informed than the vast majority of health professionals about the multidisciplinary care that should be considered in the context of their transition. What about their parents, once they are gathered in a group?

Whereas the tone of the adolescent group is most often one of humor, laughter and self-mockery, even when discussing painful situations, the parent group is less in harmony: while some trivialize their family situation, others evoke their anxieties. They sometimes burst into tears, something that has yet to happen in the adolescent group.

No parent is at the same stage. The most refractory ones have left the group after a few sessions while others have stayed until today. The range of parental positions is quite broad. Some support their child unconditionally: *“I’ve been behind my daughter since I realized that her transition was vital for her.”*; “*I’ve done everything I can to speed up the process of changing civil status”,* says Billie’s mother before adding that *“The first testosterone injection was a happy moment shared with family and friends.”* Tatiana’s mother: *“In France, it takes years to undergo surgery. And so, I’m accompanying my daughter abroad for her 18th birthday. I can’t wait for the surgery so that her “dysphoria” can be alleviated!”* Other parents will object to any form of transition: *“I won’t use her new name! My daughter will always remain a girl!”* Between these two extremes, all intermediaries are possible.

Concerns about their child’s body are recurrent: what are the post-operative side effects? How does one assess the risk of failure? *“What if he wants to reverse the operation?”* Reassignment surgeries transform from one day to the next and are irreversible. Hormone therapies, whose results appear gradually, generate equally perceptible anxiety: *“Do we have enough insight into the undesirable effects of puberty blockers or hormones on the bones?”*

Further away from the soma, the topic of “mourning the child as imagined by his parents” comes up frequently, and often with the same stages: *“First, she cut her hair. Then her birth name disappeared from the family record book. Then* he *started testosterone, and soon it will be a mastectomy. I thought I had mourned my daughter. But now, since he doesn’t want to preserve his eggs, I’m at a loss. I will never be a grandmother!”,* says Michael’s mother before she breaks down in tears. Michael’s mom has been a single parent since her son was 3 years old. She is doing her best but sometimes loses self-assurance. In order to comfort her, Daniel’s father answers: *“You might be right… However, at least, you won’t lose your son. Mine was on his fourth suicide attempt. Since he became a man, he has been living again!”.* Supporting the transition of one’s child can significantly reduce the risk of suicide (Toomey and Syvertsen, 2018).

Another recurring question for the parents of the group concerns their own personal experience since their children’s coming-out: *“For me, the news was like an atomic bomb; but in the end, it was nothing compared to our fear of suicide attempts.”* Another father: *“Christopher didn’t like dresses. I thought she would become homosexual, but not transgender. And by the way, I didn’t even know what it was at the time!”.*

For Steve’s mother – who is a single parent for her adopted child – the detonator was her encounter with a mother and her transgender daughter in the waiting room that made the difference: *“The kid looked happy and fulfilled. I found out by chance that she was transgender, despite her totally ordinary appearance,”* she recalls. *“Then I talked about trans identity with her mother, a gentle woman who was more advanced than I was, and it soothed me. It changed my mind, and I started supporting my son, especially against bullying!”.* Indeed, Steve’s mother had to go to the high school several times before coming to an agreement with the director for a “social transition” (for her son to be called by his boy’s name and to be treated as a boy). However, the school doctor decided that this would not be possible and made an announcement to all the teachers and students of the establishment reminding them that Steve was *“born a girl,”* had lied, and should henceforth be called by his female name. This situation inevitably led to Steve suddenly dropping out from school. Steve never returned to that high school again.

After about a year, the exchanges between the parents have ended up becoming more decentralized: recently, trans identity issues have given way to problems classically found in adolescence: *“If my daughter is looking for her place so much, it’s because she is going through some classic teenage rebellion!”.* After a while though, people do zoom out and are able to see the whole picture.

When the transgender teens and their parents meet for a multi-family session, nobody cuts anybody off. The parents generally have an attentive attitude and some of them are impressed and moved to watch their own child inside of a consolidated group made up of other transgender teenagers. The co-therapists try to promote therapeutic outcomes in case of psychological suffering or difficulties in family relationships by inviting the members of the group to talk about anything that comes to their mind, *“even if it’s not easy”*: *“My mother and I have been rejected from her family in Marseille. She is a lesbian and I’m trans… but we don’t really care because they are all mafiosos over-there!* (laughs). *In the beginning, my mom’ and I disagreed a lot… but now we've moved from Marseille to Paris, and we are just fine!”* Paradoxically, it’s often easier to talk about difficult issues during a multi-family group because the participants who are witnessing the verbal exchanges also constitute a very solid support (Rabain, 2018).

But why and how do multi-family sessions make it possible to discuss the challenges encountered by transgender adolescents and their parents regarding their social adaptation? How do they promote therapeutic outcomes in case of psychological suffering or difficulties in family relationships? How do they sometimes even prevent the disruption of family relations?

## Discussion

### Movements of Transference and Countertransference

The multi-family group modifies the transference movements by dispersing the projections on different members of the group. Gathering several families with co-therapists and a secretary generates “multiple transfers”. This particular transference dynamic allows the participants to avoid the negative effects of a massive dual transference. More specifically, the psychoanalytic multi-family setting generates transference elements that can be categorized in three ways. The transference can first be “radial” 1), that is, directed to the co-therapists. Then, the transference can be “horizontal” 2) when targeting other members of the group. That type of transference reinforces along the sessions thanks to the resonance effects between participants facing common issues. Some crossed identifications which consist in investing other parents of the group can be very helpful for some teenagers whose parents have narcissistic weaknesses. Indeed, multi-family groups consolidate identity and reinforce narcissistic stability. In other words, the identification games promoted by this setting supports adolescents as well as their parents when they have narcissistic weaknesses. And finally, the transference can aim the group “as a total object” 3) (Rabain and Bourvis, 2016, 320).

According to J. García Badaracco: “*The participation of a patient in a* [psychoanalytic multi-family] *group* […] *increases the work of elaboration. This work pushes the members of the family to relax their defenses.*” (2000, 66, personal translation). Whereas the transference issues are qualified as “multiple” – or “diffracted,” according to the translation – the countertransference movements in such a setting are “protean” as far as the co-therapists identify with both adolescents and their parents. Another reason why our supervision sessions have been really helpful.

### Promoting Mental Health

Our innovative mental health care approach made it possible for both the adolescents and their parents to break out of a certain form of isolation. In the adolescents’ group, regardless of the singularity of their experiences, everyone, without exception, has experienced transphobia, being rejected and ostracized, which is very common (Simons and Schrager, 2013, 791). During the first year of the discussion group, manifestations of respect and attentiveness to other group members brings to mind the phenomenon of “group illusion” as defined by Anzieu (1991), (362–363): non-conflictuality and a feeling of self-sufficiency coexist, as though all necessary models were to be found through the diversity of material supplied by the participants. As the group envelope was formed, a boundary between a good interior and a hostile exterior materialized. The cleavage between good and bad was therefore not within the group, but between the group and the exterior (Anzieu, 1975; Anzieu, 1985).

The gaze of cisgender people can sometimes be experienced as rejecting and scornful, or even curious, in a voyeuristic way (Bufnoir, 2016). To be included in a discussion group which gathers transgender adolescents is therefore a way to reinforce one’s own narcissism. It also promotes mental health by informing the members of the group of the different possibilities of attention they can receive either in the hospital or in a number of associations: transgender associations such as OUTrans^1^, associations against transphobia, LGBTIQA+ associations, etc.

In the same way, the parents also question very important themes such as suicide and survival of their child. For example, a recurring and difficult issue for the parents of the group is the “mourning of the child they had dreamed of” (Lebovici and Stoléru, 1983; Lebovici, 1998). Being together, trying to elaborate together helps not only with treating their own anxiety but also makes it possible to better position themselves in front of their child, which is essential for supporting them (Menvielle and Rodan, 2011). From a psychiatric standpoint, there is a recrudescence of signs of anxiety and depression among transgender teenagers. For these reasons, our groups can be considered as a preventative approach, although they are not limited to prevention.

### Prevention of Mental Disorders

The anxiety and depression found in transgender teenagers are linked to specific aspects of their environment (Bernard and Wathelet, 2019, 111–123). Risk factors include bullying at school or in the neighborhood and the transphobia of family members. Indeed, gender-related stigmatization increases the risk of developing a depressive syndrome fivefold compared to the general adolescent population (Kaltiala-Heino and Bergman, 2018, 31–41). Hence the importance of providing medical and psychological support to transgender minors and their parents.

Among the teenagers of our Sexual Identity Consultation, approximately 70% have anxiety issues, and more particularly an “anxiety-depressive disorder” according to the annual statistical report on the activities of our Adolescent Psychiatry Department (2019). There are also phobic reactions, in particular school phobia which is very common among transgender adolescents, leading to subjects totally dropping out of school. This phenomenon is more frequent with this group compared to the general population of our Department: the dropout rate is 38%. Faced with this data, we are discussing the possibility of a new study, with the help of other Adolescent Psychiatry Departments, in order to compare the rate of anxiety-depressive syndrome in subjects who have benefited from our mental health care approach against a control group of subjects who have not received any group support.

As soon as the group envelopes were strong enough, the first movements of differentiation could finally appear: some of them want to be considered as transgender teenagers whereas other ones just want to be considered as a boy or as a girl; that is, if one is to live happily, one must live in hiding. While other members argued that to defend themselves effectively, it was necessary to be firm. In the same way, where some were horrified by the Pride march, others said that they adored Gay Pride.

While the community environment and social networks offer a wide variety of identificatory figures to transgender teenagers, the adolescent group reinforces the identification game *in situ*. In addition to fighting against a tendency towards deadly isolation, the sessions allow for identifications and counter-identifications with binary or non-binary participants, which revive their psychic associativity. Thus, the “gender-fluid generation” – referring to the new generation of adolescents and adults in which some people feel that they are neither quite man nor quite woman – remains undeniably more flexible than the previous one, especially with regard to gender identity and the question of sexual orientation. In this group, almost every participant was involved in a romantic relationship from the beginning. However, no one would talk about it spontaneously. No one knew who was with a boy or a girl; with a cisgender or a transgender partner. After one year, the teenagers became less evasive and started to talk about their love affair. In other words, this group approach reinforces the ability to associate and redeploy libido onto objects of substitution (Rabain, 2017; Rabain, 2018). Another main goal of this group is « *to strengthen the personality – process of subjectivation – and to promote a better emotional stability with a greater capacity to face and endure traumatic events* ». (García Badaracco, 2000, (235, personal translation).

### Prevention of Disruptions of Family Relations

At first, the parents’ group was characterized by an initial distrust. Some of them thought that we were trying to convince their child to go through the transition. However, the sessions quickly led to better cooperation. While a certain ambivalence was evident during the first sessions, it progressively faded away in favor of a more secure space for discussion. The parents did not remain focused on issues limited to trans identity: they finally zoomed out, probably thanks to our support group, among other things. It is essential to work with parents for the sake of their child because the position of the parents has an obvious influence on the transition path of transgender teenagers. Helping parents to articulate their own point of view and move away from a monolithic and often normative stereotypical view of transgender issues constitutes a significant benefit for their child (Menvielle and Rodnan, 2011; Simons and Schrager, 2013). Working through parental fears and taking into account the principle of reality – for example, by not denying the many risks faced by trans people – leads them to be better able to protect their child. This preliminary work leads to an increased mutual understanding during multi-family gatherings.

Another important point: there is a logical time lag between the experience of the transgender adolescents and that of their parents. At the moment when teenagers are about to come out, they have been usually been working on it for a long time. However, by the time they finally say it, they are likely to expect their parents to react immediately. For instance, when they come out as a transgender teen, most of them already know that they want to get hormones. They have already decided to make this request since the idea has generally been there for a couple of years. Parents, on the other hand, have often not thought about such things before. They therefore cannot take a position as quickly as their child would for like them to. In this situation, the welcoming of new participants by the veteran ones can be really helpful. Beyond the mutual narcissistic revalorization generated by this activity, it would seem that the veteran parents in the group identify in the new members the distress they once experienced and, as a result, how far they have come since then. In return, the new participants have more identificatory models to rely on. This valuable relief reduces tensions within the family and helps to prevent the disruption of family relations.

Moreover, in these intergenerational multi-family groups, the narration of the parents’ point of view invites every adolescent to take an interest in their own family values and encourages the emergence or reinforcement of their own capacity for speaking up. This gives new energy to the process of subjectivation, which requires an unavoidable “clash of arms” between adolescents and their parents (Winnicott, 1971, 200). Let us make it clear here that this “confrontation between generations” (Kancyper, 2018) is a necessary although not sufficient condition for subjectivation. Indeed, any conflict, however painful it may be, does not always lead to fruitful changes. Our group approach to mental health increases the chances that confrontation between adolescents and their parents may reinforce the process of subjectivation.

## Conclusion

In this article, we discussed why we have to take care of adolescents who question their gender identity with their parents in a multi-family-oriented approach. We also question how to support their parents, who are often destabilized at the sight of a pubertal process different from the one they experienced at the same age. The group generated a dynamic that allowed its members to put their questions into perspective in the face of the experiences of other families. This resulted in a new impetus and the readjustment of certain positions hostile to transgender people.

According to the French *Trans identity Observatory* ^2^, there are more attacks against transgender women than transgender men. These usually involve serious assaults and crimes, which is particularly stressful for parents. Anxiety is often aroused by the inability to think about and talk about these questions of trans identity. In our approach, one of our main goals consists in co-constructing a whole panel of representations and signifiers that the transgender teens and their parents will be able to use to build their own representations. In other words, all roads leading to a greater number of representations will constitute a victory against the progression and permanent installation of mental health disorders.

In the future, why not add the brief intervention of transgender associations? They would likely be a great help in the work of putting into words what people feel before, during and after the transition process. Talking with transgender adults who are doing well and who are the authors of their own lives could eventually result in a significant aid for the project of destigmatizing trans identity.

## Data Availability Statement

The original contributions presented in the study are included in the article/Supplementary Material, further inquiries can be directed to the corresponding author.

## Ethics Statement

Ethical review and approval was not required for the study on human participants in accordance with the local legislation and institutional requirements. Written informed consent to participate in this study was provided by the participants’ legal guardian/next of kin.

## Author Contributions

The author confirms being the sole contributor of this work and has approved it for publication.

## Conflict of Interest

The author declares that the research was conducted in the absence of any commercial or financial relationships that could be construed as a potential conflict of interest.

## Footnotes

1
www.outrans.org.

2
https://www.observatoire-des-transidentites.com.

## References

[B1] Anzieu D. (1991). “Illusion groupale,” in Dictionnaire de psychologie. Editors Doron R. Parot F. (Paris, France: PUF), 362–363.

[B2] Anzieu D. (1975). Le groupe et l’inconscient – L’imaginaire groupal. Paris, France: Dunod.

[B3] Anzieu D. (1985). Le Moi-peau. Paris, France: Dunod.

[B4] Bernard M. Wathelet M. Pilo J. Leroy C. Medjkane F. (2019). Identité de genre et psychiatrie. Revue Adolescence. 37 (1), 111–123. 10.3917/ado.103.0111

[B5] Bufnoir J. (2016). Des embarras du psychanalyste face à l’adolescent transgenre. Revue Enfances & PSY. 69, 66–74. 10.3917/ep.069.0066

[B6] Condat A. Bekhaled F. Mendes N. Lagrange C. Mathivon L. Cohen D. (2016). La dysphorie de genre chez l’enfant et l’adolescent : histoire française et vignettes cliniques. Neuropsychiatrie de l'Enfance et de l'Adolescence. 64, 7–15. 10.1016/j.neurenf.2015.06.001

[B8] García Badaracco J. E. (1989). Comunidad terapéutica psicoanalítica de estructura multifamiliar. Madrid, Spain: Ed. Tecnipublicaciones.

[B9] García Badaracco J. E. (2000). Psicoanálisis multifamiliar. Los otros en nosotros y el descubrimiento de si mismo. Buenos Aires, Argentina: Ed. Paidós.

[B10] Kaltiala-Heino R. Bergman H. Työläjärvi M. Frisén L. (2018). Gender dysphoria in adolescence: current perspectives. Adolesc. Health Med. Therapeut. 9, 31–41. 10.2147/AHMT.S135432 PMC5841333 29535563

[B11] Kancyper L. (2018). La confrontation entre les générations. Paris, France: L’Harmattan.

[B12] Lebovici S. (1998). L’arbre de vie – Éléments de la psychopathologie du bébé. Paris, France: Erès.

[B13] Lebovici S. Stoléru S. (1983). Le nourrisson, sa mère et le psychanalyste – les interactions précoces. Paris, France: Bayard.

[B16] Mendès N. Lagrange C. (2016). La dysphorie de genre chez l’enfant et l’adolescent : revue de littérature. Neuropsychiatrie de l’enfance et de l’adolescence. 64, 240–254. 10.1016/j.neurenf.2016.04.003

[B17] Menvielle E. Rodnan L., (2011). A therapeutic group for parents of transgender adolescents. Child Adolesc. Psychiatr. Clin. N Am. 20 (4), 733–743. 10.1016/j.chc.2011.08.002 22051009

[B18] Rabain N. Bourvis N. Cohen D. (2016). Les groupes analytiques multifamiliaux pour adolescents. Neuropsychiatrie de l’enfance et de l’adolescence. 64 (5), 317–323. 10.1016/j.neurenf.2016.06.007

[B19] Rabain N. (2017). La psychanalyse multifamiliale pour adolescents à Buenos Aires. Rev. Fr. Psychanal. 81 (4), 1146–1153. 10.3917/rfp.814.1146

[B20] Rabain N. (2018). Pourquoi la psychanalyse multifamiliale ? Enfances Psy. 79, 32–39. 10.3917/ep.079.0032

[B21] Racamier P.-C. (2002). L’esprit des soins – le cadre *.* Paris, France: CPGF.

[B23] Simons L. Schrager S. Clark L. F. Belzer M. Olson J. (2013). Parental support and mental health among transgender adolescents. J. Adolesc. Health. 53 (6), 791–793. 10.1016/j.jadohealth.2013.07.019 24012067 PMC3838484

[B24] Toomey R. B. Syvertsen A. K. Shramko M. (2018). Transgender adolescent suicide behavior. Pediatrics. 142 (4), e20174218. 10.1542/peds.2017-4218 30206149 PMC6317573

[B25] Winnicott D. W. (1971). “Concepts actuels du développement de l’adolescent – leurs conséquences quant à l’éducation,” in Jeu et réalité (Paris, France: Gallimard), 190–207.

